# Deterministic fate assignment of Müller glia cells in the zebrafish retina suggests a clonal backbone during development

**DOI:** 10.1111/ejn.14257

**Published:** 2018-12-04

**Authors:** Steffen Rulands, Ana Belen Iglesias‐Gonzalez, Henrik Boije

**Affiliations:** ^1^ Max Planck Institute for the Physics of Complex Systems Dresden Germany; ^2^ Center for Systems Biology Dresden Dresden Germany; ^3^ Department of Neuroscience Uppsala University Uppsala Sweden; ^4^ Department of Physiology Development and Neuroscience University of Cambridge Cambridge UK

**Keywords:** bipolar cell, *Danio rerio*, GFAP, retinal progenitor cell, stochastic

## Abstract

The optic cup houses multipotent retinal progenitor cells that proliferate and differentiate to form the mature retina, containing five main types of neurons and a single glial cell type, the Müller cell. Progenitors of the zebrafish optic cup generate clones that vary regarding the number and types of neurons, a process we previously showed could be described by stochastic models. Here, we present data indicating that each retinal progenitor cell, in the 24 hrs post‐fertilization optic cup, is predestined to form a single Müller cell. This striking fate assignment of Müller cells reveals a dual nature of retinal lineages where stochastic mechanisms produce variable numbers of neurons while there is a strong deterministic component governing the formation of glia cells. A possible mechanism for this stereotypic fate assignment could be the maintenance of a clonal backbone during retina development, which would be similar to invertebrate and rodent cortical neurogenesis.

**Abbreviations**
AC: Amacrine Cell
BC: Bipolar Cell
GC: Ganglion Cell
HC: Horizontal Cell
hpf: hours post‐fertilization
MC: Müller cell
NB: Neuroblast
PR: Photoreceptor Cell
RPC: Retinal Progenitor Cell

## 1 INTRODUCTION

The nervous system is principally made up of neurons and glia. There are still many unanswered questions regarding how proliferation is controlled, how fates are assigned, and the potency of different progenitors. During development, multipotent retinal progenitor cells (RPCs) in the optic cup proliferate and differentiate into five types of neurons and one type of glia, the Müller cell (MC; Boije, MacDonald, & Harris, 2014; Masland, 2001). Müller cells are generated at the end of the lineage and their determination relies on the Notch‐Delta pathway (Bernardos, Lentz, Wolfe, & Raymond, 2005; Furukawa, Mukherjee, Bao, Morrow, & Cepko, 2000; Young, 1985). Time‐lapse imaging of single RPCs shows that one cell within the developing clone upregulates Notch‐signalling, maintains the radial morphology, and eventually differentiates into a MC (MacDonald et al., 2015). Mature MCs retain an expression profile and morphology similar to RPCs and display stem cell‐like properties as they can, in several species, dedifferentiate, re‐enter the cell cycle and generate neurons in response to injury (Blackshaw et al., 2004; Fischer & Reh, 2003; Wang et al., 2017).

In vitro and in vivo studies have shown that clones, generated from single RPCs, are variable regarding the number and type of neurons (Boije, Rulands, Dudczig, Simons, & Harris, 2015; Gomes et al., 2011; He et al., 2012). We previously showed that the assignment of neuronal fates in the zebrafish retina could be described by a stochastic model, where the independent expression of key fate determinants predicted the experimental clonal data (Boije et al., 2015). Since previous studies focused on the neural components, we have here undertaken blastomere transplantations, using transgenic zebrafish, to assess the formation of MCs within retinal clones. Unexpectedly, we found that individual RPCs in the 24 hrs post‐fertilization (hpf) optic cup generate exactly one MC in the vast majority of clones, independently of the overall clone size. This reveals a double nature of retinal lineages where both stochastic and deterministic traits govern clonal development.

## 2 MATERIALS AND METHODS

### 2.1 Fish maintenance

Zebrafish were maintained and bred at 26.5°C at a 14:10 hr light‐dark cycle. Embryos were raised at 25–32°C and staged based on hours post‐fertilization (hpf; Kimmel, Ballard, Kimmel, Ullmann, & Schilling, 1995). All animal work was approved by Local Ethical Review Committee at the University of Cambridge and performed according to the protocols of project license PPL 80/2198. The transgenic lines used have all been described previously; Tg*(gfap:GFP)* ^*mi2001*^ (Bernardos & Raymond, 2006), Tg*(vsx2:EGFP;* Kimura, Okamura, & Higashijima, 2006), Tg(*Ptf1a*‐dsRed*;*He et al., 2012).

### 2.2 Transplantations

Blastomere transplantations were performed as described in Boije et al. (2015). Briefly, high‐ to oblong‐stage embryos were dechorionated by pronase digestion (Sigma), placed in agarose molds, and between one to five blastomeres were transferred between embryos using a glass capillary connected to a 2 ml syringe. At 24 hpf, embryos were anaesthetized by 0.04% MS‐222 (Sigma), screened on an upright fluorescent microscope where isolated GFP‐positive RPCs could be identified. At 72 hpf, embryos were fixed for 1 hr in 4% PFA, the eye dissected out, and mounted in 1% low melting agarose (Sigma) for imaging.

### 2.3 Morpholino injections

Antisense translation blocking morpholinos were obtained from Gene Tools, reconstituted as 1 or 3 mM stock solutions in water, and injected into the yolk at the one‐cell stage. Morpholinos targeting Ptf1a, Atoh7, and Vsx1 have all been described previously and sequences are listed in Boije et al. (2015).

### 2.4 Imaging

Embryos were treated with 0.003% phenylthiourea (Sigma) from 10 hpf to prevent pigmentation. Retinal clones were imaged under 60× (NA = 1.30) or 30× (NA = 1.05) silicon oil objectives on an inverted laser‐scanning confocal microscope (Olympus FV1000) fitted with GaAsP detectors.

## 3 RESULTS

### 3.1 Each RPC at 24 hpf seems predestined to give rise to a single MC

**Figure 1:**
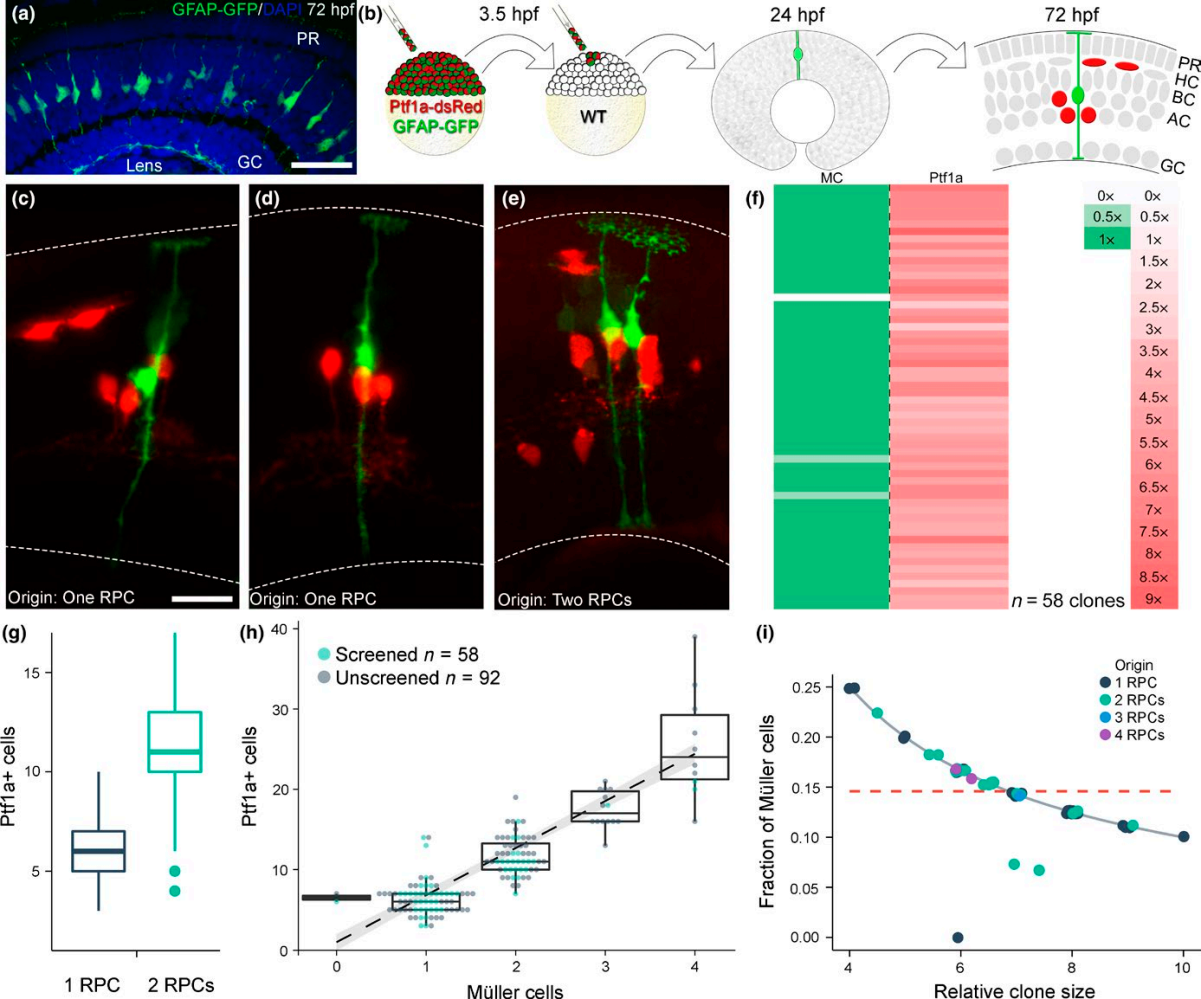
Correlation between retinal progenitor cells and Müller cells. (a) Sections of GFAP‐GFP transgenic retinas were counterstained with DAPI and quantified. (b) Transgenic blastomeres were transplanted into wild‐type embryos to generate labelled clones. (c–e) Clones originating from one or two RPCs, scored at 24 hpf, and imaged at 72 hpf. Dashed lines mark the limits of the retina. (f) Clones generated by RPCs scored at 24 hpf normalized to the number of start RPCs. (g) Distribution of Ptf1a lineage in clones originating from one or two RPCs. (h) Correlation between Müller cells and Ptf1a‐positive cells within a clone. (i) The fraction of MCs compared to the clone size (i.e., MCs/(MCs+Ptf1a‐positive cells)) follows the curve expected for a deterministic fate assignment rather than the one expected by a stochastic model (dotted line). Scale bar represents 20 μm in a and 10 μm in c–e. [Colour figure can be viewed at wileyonlinelibrary.com]

Quantification of MCs in the differentiated central region of the 72 hpf zebrafish retina revealed a ratio of one in 22.6 ± 2.4 between GFAP‐positive cells (i.e., MCs) and DAPI (4.4% of 6,330 cells counted in 13 sections from seven fish), well in line with proportions reported in other vertebrate retinas (Young, 1985; Figure 1a). Strikingly, we previously showed that the average size of a clone generated by a 24 hpf RPC is 22.3 ± 4.1 cells, indicating a link between the progenitors of the undifferentiated optic cup and the number of MCs in the mature retina (Boije et al., 2015).

Clonal analysis was performed by transplanting blastomeres from GFAP‐GFP, Ptf1a‐dsRed double transgenic embryos into wild‐type embryos, followed by a screen at 24 hpf and imaging at 72 hpf (Figure 1b). The GFAP‐GFP transgenic line expresses low levels of GFP in RPCs at 24 hpf allowing the screen for isolated transgenic cells in the host optic cup following transplantation (Bernardos & Raymond, 2006). The columnar clones formed by one to four transplanted RPCs were analysed at 72 hpf, at which time GFAP is restricted to MCs and Ptf1a is expressed in amacrine and horizontal cells (ACs and HCs; Bernardos & Raymond, 2006; Jusuf et al., 2011). There is a strong correlation between the number of RPCs scored at 24 hpf and the number of MCs at 72 hpf (*p* = 2.2e‐16, Spearman's rank correlation), with a one‐to‐one ratio in the majority of clones (55 of 58; Figure 1c–f, Table S1). The distribution of Ptf1a‐positive cells in this study (6.2 ± 2.0 and 11.3 ± 3.2, originating from 1‐RPC and 2‐RPCs respectively) was close to identical to the numbers described in Boije et al. (2015) (6.2 ± 1.4 and 11.2 ± 2.0). Despite being variable, the number of Ptf1a‐positive cells follows a bimodal distribution (*p* = 4e‐5, Hardigan's dip‐test), allowing the initial number of labelled RPCs to be inferred from the number of Ptf1a‐positive cells (Figure 1g). A further 92 unscreened clones were generated, which were quantified for Ptf1a‐ and GFAP‐positive cells at 72 hpf. These group well with the data from the screened clones and emphasize the strong correlation between the number of Ptf1a‐positive cells and the number of MCs (Figure 1h, *p* = 3.3e‐32). Of the 150 clones quantified, 68 screened/predicted to have originated form a single RPC, only two lack MCs, and no clones host more MCs than expected from the number of start RPCs.

While a correlation between the number of MCs and the number of Ptf1a‐positive cells is expected also in the event of purely stochastic fate assignment, as long as the ratios of cell types are globally controlled, we asked whether the low amount of variability in this correlation might indicate a deterministic component in MC fate assignment. To test this, we considered a situation where the generation of MCs is regulated by a stochastic and independent fate assignment. In this case, the fraction of MCs among all quantified cells (i.e., MCs/(MCs+Ptf1a‐positive cells) would be independent of the total number of quantified cells (dashed line in Figure 1i). By contrast, if the assignment of MC fate would be deterministic, the fraction of MCs would be inversely proportional to the total number of quantified cells (solid line in Figure 1i). We can use this distinction in the scaling of the fraction of MCs to test if the clonal data are in agreement with stochastic fate assignment using Spearman's rank correlation. We found that for the vast majority of clones the fraction of MCs follows the pattern expected for deterministic fate choice, arguing strongly against a stochastic and independent fate assignment (*p *< 2.2e‐16).

### 3.2 Knock‐down of Ptf1a and Atoh7 affects the formation of MCs

As MCs were not analysed in our previous clonal study, focused on neural fate determination, we wished to complement these results by investigating the fate assignment of MCs in clones where morpholinos are used to knock down key fate influencing transcription factors; Atoh7 for ganglion cells, Ptf1a for ACs and HCs, and Vsx1 for bipolar cells (BCs; Boije et al., 2015). We refer to our previous study for a thorough description of the minimal effect the different morpholinos have on the number of Ptf1a‐positive neurons and overall clone size (Boije et al., 2015). Notably, Ptf1a morphant clones contain the same amount of cells labelled by the Ptf1a‐dsRed construct as control clones since the cells retain the expression of the fluorophore despite the fate‐shift to other cell types.

Morpholinos were injected at one‐cell stage in GFAP‐GFP, Ptf1a‐dsRed double transgenic embryos, followed by blastomere transplantation into wild‐type hosts and analysis at 72 hpf. Here, the number of Ptf1a‐positive cells was used to infer the number of start RPCs.

**Figure 2:**
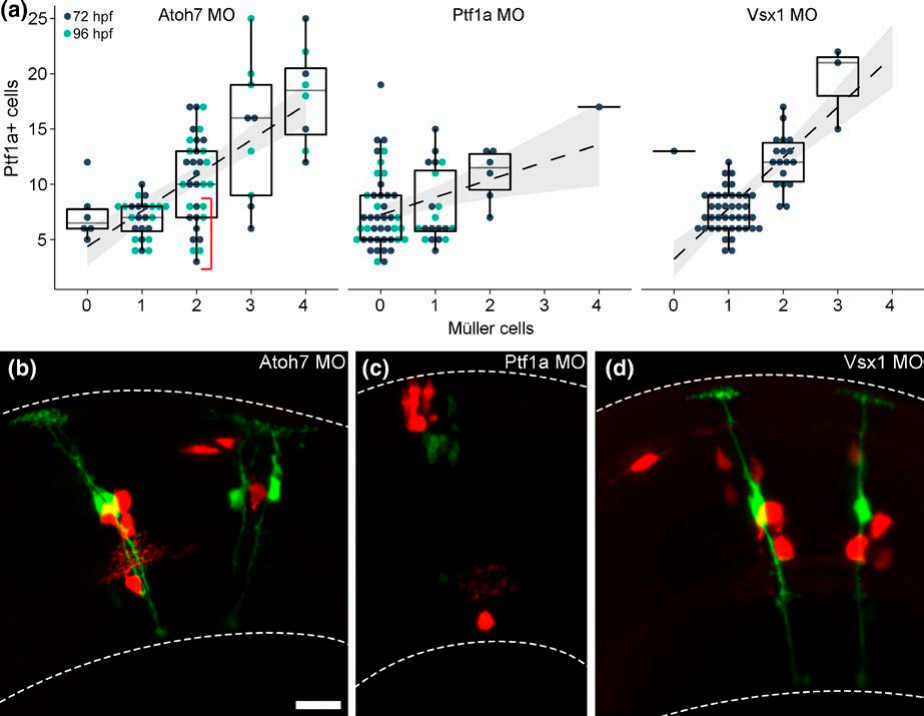
Formation of Müller cells in morphant clones. (a) Clone data where Atoh7, Ptf1a, or Vsx1 was knocked down using morpholinos. Data display the correlation between Müller cells and Ptf1a‐positive cells within a clone. Red bracket in Atoh7 morphant graph features a cluster of clones hosting two MCs predicted to have originated from a single RPC. (b) Two Atoh7 morphant clones, containing two MCs each, predicted to have originated from single RPCs based on the number of Ptf1a‐positive cells. (c) A Ptf1a morphant clone where the number of fate‐shifted red cells suggests that the clone originated from a single RPC. (d) Two Vsx1 morphant clones. Dashed lines mark the limits of the retina. Scale bar in b represents 10 μm and is also valid for c and d. [Colour figure can be viewed at wileyonlinelibrary.com]

While knock‐down of Vsx1 did not affect the formation of MCs, there was a drastic effect when morpholinos targeting Ptf1a and Atoh7 were injected (Figure 2a–d). Clones where Ptf1a was knocked down showed a significant loss of MCs (43 out of 70 clones had no MCs; Figure 2a,c). The correlation between MCs and Ptf1a‐positive cells was significantly different from the wild‐type clones (ANOVA, *p* = 2.9e‐7). Atoh7 morphant clones showed a dual phenotype; six clones had no MCs while others displayed more MCs than predicted (15 out of 80 clones; Figure 2a,b, note lower cluster for 2 MCs in graph). These Atoh7 morphant clones represent the only occasion where the number of MCs was in excess compared to the predicted number of origin RPCs. There was a significant difference in the correlation between MCs and Ptf1a‐positive cells in Atoh7 morphant clones compared to wild‐type clones (*p* = 0.04). Clones analysed at 96 hpf, to exclude developmental delay in morphant clones, showed the same distribution as clones analysed at 72 hpf (Figure 2a).

### 3.3 Vsx2‐reporter reveals a correlation between MCs and BCs

**Figure 3:**
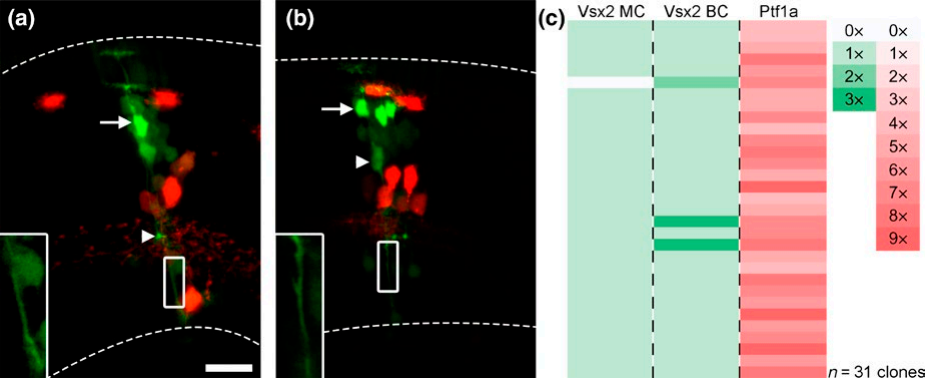
Correlation between the Ptf1a lineage and the Vsx2 lineage. (a) Clone generated from a Ptf1a‐dsRed, Vsx2‐GFP double transgenic RPC. Arrow indicates the soma of the bipolar cell (BC) and the arrowhead its synaptic button. Boxed region is magnified and depicts the single Müller cell (MC) process. Dashed lines mark the limits of the retina. (b) Example of clone with three Vsx2‐positive BCs and one MC. Arrow indicates BCs, arrowhead indicates the MC soma, and the single process is shown magnified in the boxed region. (c) List of Vsx2‐GFP, Ptf1a‐dsRed double transgenic clones normalized to the predicted number of start RPCs. Scale bar in a represents 10 μm and is also valid for b. [Colour figure can be viewed at wileyonlinelibrary.com]

To verify our results regarding MCs, we used a Vsx2‐reporter line, known to label MCs and one subtype of BCs (Vitorino et al., 2009), to perform blastomere transplantations. Clones from Vsx2‐GFP, Ptf1a‐dsRed double transgenic embryos were analysed at 72 hpf where Vsx2‐positive BCs can be distinguished by their somal position, their strong expression of GFP, and their synaptic button in the inner plexiform layer, while MCs, express GFP weaker and extend processes spanning the width of the retina (Figure 3a). All but one of the 31 clones predicted to have originated from a single RPC, based on their number of Ptf1a‐positive cells, had a single MC (Figure 3c). Interestingly, most clones also hosted a single Vsx2‐positive BC (Figure 3a,c). Notable is that the clone lacking a MC hosted two Vsx2‐positive BCs. Two of the clones, hosting a single MC, had three Vsx2‐positive BCs (Figure 3b,c).

## 4 DISCUSSION

While stochastic modelling can recapitulate the neural variability in clones generated by individual RPCs, less is known regarding the glial component (Boije et al., 2015; Gomes et al., 2011; He et al., 2012). Here, clonal analysis in the zebrafish retina, focused on glia cells, revealed an unexpectedly tight correlation between RPCs and MCs. Our data suggest that each RPC in the undifferentiated 24 hpf optic cup is predestined to give rise to one, and only one, MC.

### 4.1 Clonal analysis of retinal progenitor cells

Besides the striking similarity between the average size of a clone generated by a 24 hpf RPC (22.3 cells, Boije et al., 2015) and the ratio of MCs in the differentiated retina (1 in 22.6 cells), we also show, through clonal analysis, a strong correlation between the RPCs of the optic cup and MCs in the mature retina. The analysis is unique in that our clones invariably originate from RPCs of the 24 hpf optic cup, a homogeneous group of quiescent progenitors, prior to the onset of proliferation and differentiation (Hu & Easter, 1999). The clonal dataset reveals that each of the 24 hpf RPCs consistently forms a single MCs, a process clearly not governed by a stochastic process (Figure 1i). The distribution of Ptf1a‐positive cells in these clones were close to identical to our previous study, where we used the neutral marker H2B‐GFP, validating the screening strategy and the clonal analysis (Boije et al., 2015). Although cell death is not a major factor during zebrafish retina development, 1%–2% of the cells undergo apoptosis (He et al., 2012), which may explain the few clones lacking MCs in our study.

Turner and Cepko (1987) quantified almost 3,000 clones in the rat retina and while most of them, some 2,100, contained only rod PRs, there were 112 clones that contained glia. Interestingly, all of these hosted a single MCs, born alongside either rod PRs and/or BCs, indicating that there are no gliogenic divisions in the retina. In a follow‐up study, the 42 (out of 667) mouse retinal clones containing glia, all had a single MC (Fields‐Berry, Halliday, & Cepko, 1992). Lineage reconstruction of clones generated by cultured single rat RPCs showed that the 13 (out of 129) lineages that contained glia all hosted a single MC, which was formed at the end of the lineage together with rod PRs or BCs (Gomes et al., 2011). Clonal analysis in the zebrafish retina scored 28 out of 396 clones to contain MCs, 26 of these contained a single MC (He et al., 2012). While the other two clones may represent real discrepancies in MC fate assignment, thus contradicting the absence of gliogenic divisions in the retina, it is possible that these irregularities are caused by technical limitations in the experimental procedure that made MCs difficult to identify and distinguish from BCs. Combined, these studies support our finding that RPCs only have the competence to form a single MC. However, the experimental approaches in these studies mean that the competence of the progenitor is unknown, resulting in high variability in size and composition of clones, which explain the lack of MCs in the majority of clones.

### 4.2 Effect of Ptf1a and Atoh7 on gliogenesis

Pro‐neural transcription factors, such as Ptf1a and Atoh7, are not expressed in MCs and their misexpression promotes neural differentiation at the expense of MC (Brown, Patel, Brzezinski, & Glaser, 2001; Dullin et al., 2007). Interestingly, we found that the formation of MCs was affected when Ptf1a or Atoh7 was knocked down, where a large portion of Ptf1a morphant clones failed to generate MCs and a number of Atoh7 morphant clones generated more MCs than expected.

Notch is critical for retinal gliogenesis, it maintains early progenitors in a proliferative, undifferentiated state, and later promotes the formation of MCs (Bernardos et al., 2005; Furukawa et al., 2000; Scheer, Groth, Hans, & Campos‐Ortega, 2001). Elevated Notch‐signalling in the zebrafish retina inhibits neuronal differentiation, resulting in excess and premature production of MCs, while inhibition of Notch‐signalling results in retinas lacking MCs (Randlett et al., 2013; Scheer et al., 2001).

Atoh7 promotes cell cycle exit and mouse null retinas have an increased number of cycling progenitors, where a few mutant Atoh7‐lineage cells adopt MC fate (Feng et al., 2010). Birth‐dating studies suggest that these MCs are born as early as E11.5 (Le, Wroblewski, Patel, Riesenberg, & Brown, 2006). The transcription factor p27 aids in cell cycle exit of newly formed GCs but is also continuously expressed in MCs where it plays a role in their determination (Le et al., 2006; Levine, Close, Fero, Ostrovsky, & Reh, 2000; Ohnuma, Philpott, Wang, Holt, & Harris, 1999). Hence, p27, in the absence of Atoh7, may result in the erroneous assignment of glial fate. There is also a feed‐back loop between Atoh7 and Notch, where Atoh7 activates Hes5.3, which may disrupt the delicate balance during lateral inhibition (Chiodini et al., 2013).

Transcriptional regulation of DLL1 by Ptf1a is critical for Notch‐mediated maintenance of stem/progenitor cells during pancreas development (Ahnfelt‐Rønne et al., 2012). DLL1 is required, and sufficient, to maintain progenitor cells in the retina, where it serves to activate/maintain Notch‐signalling in neighbouring progenitors to prevent their differentiation (Rocha, Lopes, Gossler, & Henrique, 2009). The loss of Ptf1a, and thereby DLL1, in the surrounding retinal cells may therefore affect the prospective MC's ability to maintain sufficient Notch levels, resulting in neurogenesis. Ptf1a forms a complex with RBP‐J, a transcriptional actuator of Notch, which activate expression of Hes1 and Hes5 by binding NICD (Beres et al., 2006; Kageyama & Ohtsuka, 1999). Ptf1a competes with NICD for RBP‐J in the mammalian spinal cord, promoting a neural differentiation programme (Beres et al., 2006; Henke et al., 2009). Loss of RBP‐J in the retina disrupts the maintenance of RPCs and increases neural differentiation (Riesenberg, Liu, Kopan, & Brown, 2009). Hence, the loss of Ptf1a may interfere with normal regulation of lateral inhibition resulting in fewer MCs being born as fate is skewed towards neurogenesis.

The altered fate assignment, and thereby changed Notch‐signalling, in Atoh7 and Ptf1a morphant clones highlight the importance of lateral inhibition controlling the formation of MCs during development.

### 4.3 Correlation between RPCs and the Vsx2 lineage

We corroborated the correlation found between origin RPCs and MCs using the Vsx2‐transgenic line. Unexpectedly, we also found a one‐to‐one ratio between MCs and Vsx2‐positive BCs, where most clones hosting a single MC also had a single Vsx2‐positive BC. Vitorino et al. (2009) showed that the two daughters of a Vsx2‐positive cell could be a BCs and a MC. Our data support these findings and suggest that the division giving rise to the MC routinely generates a Vsx2‐positive BC. In rodents, where MCs are born alongside a rod PR or a BC, asymmetrical distribution of Numb causes a division where the cell with the highest Notch levels adopts glial fate while the other becomes a neuron (Cayouette, Whitmore, Jeffery, & Raff, 2001; Gomes et al., 2011; Kechad et al., 2012). Although there might be species‐specific differences regarding the asymmetric divisions giving rise to MCs, our data suggest that Vsx2‐positive BCs may be the sister cells to MCs in the developing zebrafish retina. Further analysis through time‐lapse imaging could confirm this hypothesis.

### 4.4 A model for retinal lineages in zebrafish

**Figure 4:**
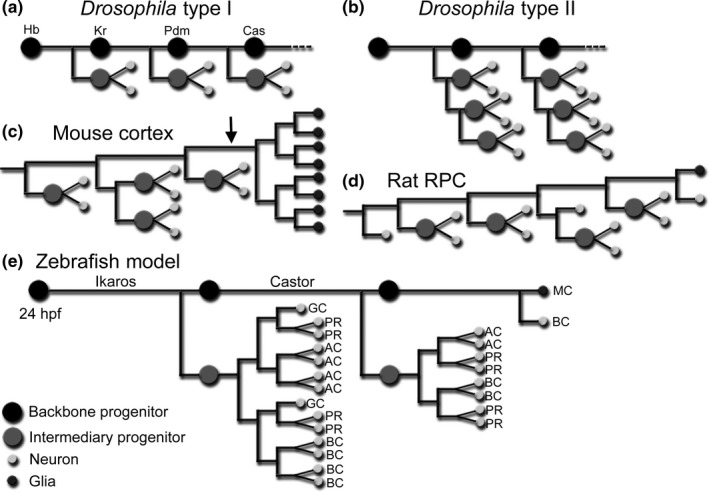
A model for retina development. (a) *Drosophila* NBs undergo self‐renewing divisions where they generate a ganglion mother cell that divides to give rise to two neurons or glia. A fate‐specifying temporal cascade changes the competence of the NB. (b) *Drosophila* Type II NBs display intermediate progenitors. (c) Mouse cortical lineages reveal a backbone where radial glia generate intermediate progenitors that proliferate and differentiate into neurons. A gliogenic switch, marked by the arrow, is followed by rapid symmetric divisions generating the glia cells (adapted from Qian et al., 2000). (d) Reconstructed lineage of a rat RPC displays intermediate progenitors that proliferate and generate neurons. At the end of the lineage a MC is born alongside a neuron (adapted from Gomes et al., 2011). (e) Model of zebrafish retina development where a backbone progenitor, kept unique by Notch, generates intermediate progenitors while transitioning through different temporal competence windows. The lineage trees of the intermediate progenitors are adapted from He et al. (2012)

There is an astounding degree of conservation of the basic processes regulating temporal competence and the maintenance of stem cells between invertebrates and vertebrates. *Drosophila* neuroblasts (NBs) undergo cell divisions where they self‐renew, retaining a backbone though asymmetric distribution of components of the Notch pathway, and bud off ganglion mother cells that divide to generate a pair of differentiated cells (Figure 4a; Bowman et al., 2008; Wang et al., 2006). Meanwhile, the NBs undergo a time‐dependent change in competence by sequential expression of regulatory transcription factors, such as *Hunchback* and *Castor*, dictating fate assignment (Isshiki, Pearson, Holbrook, & Doe, 2001). In the mammalian cortex, time‐lapse microscopy suggests the existence of a backbone, similar to that in *Drosophila* type II NBs (Figure 4b,c; Bayraktar & Doe, 2013; Costa, Bucholz, Schroeder, & Götz, 2009; Qian et al., 2000; Shen, Zhong, Jan, & Temple, 2002). Cortical radial glia produce intermediate progenitors, which in turn generate two neurons or two new intermediate progenitors, a process reliant on the Notch pathway through asymmetric segregation of Numb (Reillo, de Juan Romero, García‐Cabezas, & Borrell, 2011; Shen et al., 2002; Wang, Tsai, LaMonica, & Kriegstein, 2011). The *Hunchback* orthologue, *Ikaros*, and *Castor* were found to specify early and late fates respectively in the vertebrate cortex and retina, revealing a partly conserved temporal cascade (Alsiö, Tarchini, Cayouette, & Livesey, 2013; Elliott, Jolicoeur, Ramamurthy, & Cayouette, 2008; Mattar, Ericson, Blackshaw, & Cayouette, 2015). Following production of neurons in the cortex, the radial glia cells switch to generate glia (Costa et al., 2009; Noctor et al., 2002).

The finding that all RPCs at 24 hpf generate one, and only one, MC suggests that these represent the last equipotent progenitors and that there is a deterministic trait during retina development. We suggest that there exists a backbone progenitor during retina development, an RPC kept unique by high Notch levels, from which intermediary progenitors are produced, which proliferate and differentiate. Time‐lapse imaging of zebrafish RPCs reveals clones that fit well with the division pattern, size, and fate distribution to represent intermediary proliferating progenitor cells (He et al., 2012). We propose a model where the temporal transitions, undertaken by the backbone, are inherited by intermediary progenitors, which then abide to stochastic fate assignment for that competence window. Once the last intermediary progenitor has been produced, the backbone progenitor undergoes a final division generating the MC (Figure 4e).

## CONFLICT OF INTEREST

The authors declare no competing or financial interests.

## AUTHOR CONTRIBUTIONS

H.B. designed and performed the experiments. S.R. performed the data analysis. A.I quantified MCs in sections. H.B. and S.R. contributed to the writing of the manuscript.

## Data Availability

All clonal data can be found in Table S1.
