# Supplementary material for: Deterministic fate assignment of Müller glia cells in the zebrafish retina suggests a clonal backbone during development
Source: Eur J Neurosci. 2018 Dec 4;48(12):3597–605. doi: 10.1111/ejn.14257 (PMC6588021; doi:10.1111/ejn.14257)
Supplement: Supplementary file 2 [file EJN-48-3597-s002.docx]

Deterministic fate assignment of Müller glia cells in the zebrafish retina suggest a clonal backbone during development

Boije, Henrik ; Rulands, Steffen; Iglesias Gonzalez, Ana

_____________________________________________________________________________________

| Review timeline: | Submission date: 29/06/18  Editorial Decision: Reject and Resubmit  Revision Received: 12/09/18  Accepted: 26/10/18 |  |
| --- | --- | --- |
|  |  |  |

Editor: Paul Bolam

Reviewer 1: Anonymous

Reviewer 2: Mikiki Nagashima

Reviewer 3: David Hicks

_____________________________________________________________________________________

1st Editorial Decision	03/09/18

_____________________________________________________________________________________

Deterministic fate assignment of Müller glia cells in the zebrafish retina suggest a clonal backbone during development 

Dear Dr. Boije, 

First of all, apologies for the time it has taken to deal with your manuscript.  It has now been reviewed by two external reviewers as well as by the Editors.  Based on these reviews, we regret to inform you that we are not able to accept your manuscript for publication in EJN in its present form. However, the research described in your manuscript is potentially of interest and therefore we invite you to resubmit a much revised version. 

As you will see below, although reviewer 1 has only minor comments on the paper, reviewer 2 raises a series of rather critical concerns about the use of the same reporter (Tg(GFAP::GFP) for Muller glia cells that is used use to screen for isolated clones and the use of Ptf1a to estimate the clone size and the initial number of labeled RPCs.  Please carefully address each of the points that they have raised in the re-submitted version.   

If you do decide to re-submit, we note the following additional points that will be need to be addressed in the re-submitted version. 
- Please put the paper into EJN format with methods after the introduction and replace the Summary with a more comprehensive Abstract. 
- Include a running title, and figure/word count on the title page. 
- Include a data statement and list of abbreviations. 
- We will probably need higher resolution figures for publication.   

When revising the manuscript, please embolden or underline major changes to the text so they are easily identifiable and please don't leave 'track change' formatting marks in your paper. If the changes made are extensive, please also provide an unmarked version. 

Please provide a detailed response to the reviewers' comments, describing the changes you have made in the text. Please upload this response letter as a separate Word (.doc) file using the file designation "Authors' Response to Reviewers" when uploading your manuscript files. 

When finalized, please upload your complete revised manuscript onto the website as a Word (.doc or .docx), or .rtf file.  Please also ensure that a complete set of tables and figures is included as separate files, even if these have not changed from the originals.  At this stage it is necessary to provide high resolution figures.  Please see important instructions below. 

To re-submit your manuscript, please go to your Author Centre and check in 'Manuscripts with decisions' under the previous manuscript number for a 'Create a resubmission' link.  Also, please note that only the Author who submitted the original version of the manuscript should submit a revised version. 

If you feel that your paper could benefit from English language polishing, you may wish to consider having your paper professionally edited for English language by a service such as Wiley’s at http://wileyeditingservices.com. Please note that while this service will greatly improve the readability of your paper, it does not guarantee acceptance of your paper by the journal. 

Thank you for submitting your work to EJN. 

Best wishes, 

Paul Bolam & John Foxe 
co-Editors in Chief, EJN 


Reviews: 

Reviewer: 1 

Comments to the Author 
The manuscript, “Deterministic fate assignment of Muller glia cells in the zebrafish retina suggest a clonal backbone during development”, by Rulands et al., seeks to address the fundamental question of how the glial fate is assigned from a multipotent progenitor cell. The data provides convincing evidence that a single retinal progenitor gives rise to a single Muller glial cell, indicating that retinal progenitor fate is predetermined to be a Muller glial cell during zebrafish retinal development. This is consistent with invertebrate neuroblast and rodent cortical neurogenesis that processes relay on the activity of Notch signaling. Experiments are elegantly carried out using blastmere transplantation with transgenic lines. However, there are few (mostly minor) issues that need to be addressed to improve the manuscript. 


Specific comments 

1. Figure 2 needs more explanation of morpholino effects on overall clone size, Ptf1a+ neuron generation, and fate change of Ptf1a+ neuron in ptf1a morpholino described in the Boije et al., 2015. This is critical background information. For instance, it is imagined that knock down of Ptf1a will result in reduction of the number of Ptf1a+ neurons, which will affect correlation between MCs and Ptf1a+ neurons. Without explaining the fate change of Ptf1a neurons, the reader will hard time understanding the data. Also, it is not clear which cluster has the higher population of MCs in Atoh7 morpholino group in Fig. 2A. Have you done statistics for this?   

2. Fig. I is difficult to understand. How was the fraction of Muller cells calculated to be 0.15 if Muller glial fate is assigned by a stochastic model? How is relative clone size plotted? Supplemental table shows clone #57 and #58 both generate 4 MCs from 4 RPCs produce 20 and 21 ptf1a+ neutrons, respectively. If this is the case, should the relative clone size be 5 (20/4) and 5.25 (21/4)? Authors need to explain more details in text. 


Minor Comments: 

1. Page 4: “five clones had no MCs…” Supplemental table (page 7) shows total six clones (clone#1-6) have no MCs. 

2. Figure 1 C-D: There is no scale bar in figures. 

3. Figure 2A: Different color of dots need to be explained (72 vs 96 hpf). 

4. Allele name of the glial transgenic line from Bernardos et al., 2006 should be Tg(gfap: GFP)mi2001, not Tg(gfap: GFP)mi6 unless authors use a different line for the transgene. 


Reviewer: 2 

Comments to the Author 
The study of Rulands et al. reports that Müller glia fate assignment during retinal development is stereotypic even though they arise from lineages that are highly variable in size and neuronal composition. While the findings of this study are potentially interesting; at the current state, this manuscript does not meet the technical standards common in the field. In the current version it is not possible to appraise the data due to lack of important controls. 

The major issue is that the authors use the same reporter line to identify Muller glia cells that they use to screen for isolated clones (Tg(GFAP::GFP) in the host embryo after transplantation. However, the use of a dynamically regulated promoter is not suitable for identification of clones since it is not possible to distinguish cells of the host embryo from cells of the donor embryo in which the promoter is inactive. For such lineage analysis, the authors should use constitutively active promoters or photoconversion-based lineage analysis as beautifully done by He et al., in 2012.  Another possibility would be to use a ‘neutral’ marker like H2B or ras to label all transplanted cells in combination with the Müller glia marker. 

Another issue is that the authors estimate the clone size and the initial number of labeled RPCs in the unscreened clones based on the number of Ptf1a positive cells. This inference is crucial for all the downstream analysis in the WT and knockdown experiments. However, this reporter line only labels two cells types (amacrine and horizontal cells) that correspond to less than 30% of the total number of cells in the retina. Taking into consideration that the clones are highly variable in neuronal composition, it is not clear how the authors distinguish the technical variability (eg. number of cells labeled) from the intrinsic variability of the system to make such inferences in single clones. Moreover, it has been shown that the endpoints of the lineages are highly biased toward particular outcomes (Boije et al, 2015). Therefore, to test if the Müller glia fate assignment is in agreement with deterministic or stochastic models, the authors should have used a constitutively active reporter, as noted above, that labels the entire backbone of the lineage instead of a fraction of it that might be biased. Such a bias might explain the high prevalence of Muller glia in their analysis (148 out of 150 clones) when compared to previous lineage analysis performed (28 out of 396 clones) (He et al., 2012). 

The current manuscript is poorly written. For example, the authors did neither motivate nor conclude on the knockdown experiments. Further, considering that the results part is only three pages long, the discussion (and to some extend also introduction) seems extensive, mentioning many aspects that are not part of the current study.  The same is true for Figure 4 which is only marginally related to the presented data.

------------------------

____________________________________________________________________________________

Authors’ Response	12/09/18

_____________________________________________________________________________________

Response to reviewers:

Reviewer: 1

1. Figure 2 needs more explanation of morpholino effects on overall clone size, Ptf1a+ neuron generation, and fate change of Ptf1a+ neuron in ptf1a morpholino described in the Boije et al., 2015. This is critical background information. For instance, it is imagined that knock down of Ptf1a will result in reduction of the number of Ptf1a+ neurons, which will affect correlation between MCs and Ptf1a+ neurons. Without explaining the fate change of Ptf1a neurons, the reader will hard time understanding the data. Also, it is not clear which cluster has the higher population of MCs in Atoh7 morpholino group in Fig. 2A. Have you done statistics for this?  

*Response: There may indeed be an effect on clone size when morpholinos are administered. We showed in the Boije et al. (2015) that this is only the case for Atoh7 where we see a small increase. This is explained both in Boije et al. (2015) and in the He et al. (2012) paper. However, we have included a statement clarifying this in the current manuscript. Regarding the Ptf1a+ neurons in the knock-down; while it is true that the number of horizontal cells and amacrine cells generated through this lineage are lost the number of cells labelled red by the Ptf1a promoter driving dsRed is unchanged. This is caused by the fate-shift to other cells, rather than continued proliferation or apoptosis. This is quantified in Boije et al. (2015) and thus the correlation is between dsRed positive cells and MCs is not affected. We have emphasized this fact to make this clearer. Regarding the cluster for the Atho7 morphant group in Fig. 2A we have now marked the region in the figure discussed in the text. We did check this group for bimodality but it was not statistically significant. However, it is this group, and the other small clusters seen for 3 and 4 MC clones, that makes the correlation line statistically different from the WT graph. We have clarified this in the revised version.*

2. Fig. I is difficult to understand. How was the fraction of Muller cells calculated to be 0.15 if Muller glial fate is assigned by a stochastic model? How is relative clone size plotted? Supplemental table shows clone #57 and #58 both generate 4 MCs from 4 RPCs produce 20 and 21 ptf1a+ neutrons, respectively. If this is the case, should the relative clone size be 5 (20/4) and 5.25 (21/4)? Authors need to explain more details in text.

*Response: In a stochastic model where fate decisions are statistically independent, the number of cells of a given type in a given clone is proportional to the overall size of the clone (i.e. the fraction of this cell type is independent of the clone size). In this hypothetical case the expected (statistically speaking the maximum likelihood) fraction of MCs is given by the empirical average over all clones which is 0.15. If, however, as proposed here, this cell type follows originates from a deterministic program that is independent of the remainder of the lineage, the fraction of MCs is inversely proportional to the overall clone size. We are not doing the opposite, i.e. averaging the number of Ptf1a positive cells for a MC. Looking at the clones suggested by the reviewer we then get 4/(4+20)=0.17 and 4/(4+21)=0.16 respectively. We have clarified this in the text.*

Minor Comments:

1.       Page 4: “five clones had no MCs…” Supplemental table (page 7) shows total six clones (clone#1-6) have no MCs.

*Response: We have corrected this mistake.*

2.       Figure 1 C-D: There is no scale bar in figures. 

*Response: We have added scale bars to these figures.*

3.       Figure 2A: Different color of dots need to be explained (72 vs 96 hpf).

*Response: We have added the legend explaining this.*

4.       Allele name of the glial transgenic line from Bernardos et al., 2006 should be Tg(gfap: GFP)mi2001, not Tg(gfap: GFP)mi6 unless authors use a different line for the transgene. 

*Response: We have corrected this mistake.*

Reviewer: 2

● This manuscript does not meet the technical standards common in the field. In the current version it is not possible to appraise the data due to lack of important controls. The major issue is that the authors use the same reporter line to identify Muller glia cells that they use to screen for isolated clones (Tg(GFAP::GFP) in the host embryo after transplantation. However, the use of a dynamically regulated promoter is not suitable for identification of clones since it is not possible to distinguish cells of the host embryo from cells of the donor embryo in which the promoter is inactive. For such lineage analysis, the authors should use constitutively active promoters or photoconversion-based lineage analysis as beautifully done by He et al., in 2012.  Another possibility would be to use a ‘neutral’ marker like H2B or ras to label all transplanted cells in combination with the Müller glia marker.

*Response: While the reviewer raise an important issue regarding the use of transgenic reporter lines in research I completely disagree with the reviewer’s conclusion.*

*Firstly, regarding the transgenic zebrafish line used, GFAP-GFP. GFAP has been thoroughly described and its promoter has been used frequently to visualize MCs in both mice and zebrafish (Kuzmanovic et al. 2003. GFAP promoter drives Müller cell-specific expression in transgenic mice. Invest Ophthalmol Vis Sci. 44: 3606-13; Bernardos et al., 2006. GFAP transgenic zebrafish. Gene Expression Patterns 6: 1007–1013). The zebrafish paper by Bernados et al. (2006) has been cited 163 times according to Scopus. In the original paper a co-labeling with GFAP antibodies was performed and “All Müller processes labeled with antiGFAP also show GFP expression” where “all Müller glia in the neural retina were strongly labeled with GFP, including their radial processes, cell bodies in the inner nuclear layer…”. Numerous papers use this transgenic line to label “all Muller cells in the zebrafish retina” showing that it is an established line for visualizing MCs. Many papers have undertaken co-labeling with GFAP and other MC marker to verify the GFAP-GFP transgenic line (Koke et al. 2010. BMC Research Notes 3(50); Collery et al. 2008. Investigative Ophthalmology and Visual Science 49 (9)). In a study by MacDonald et al., (2015, J. Cell Biol. 7: 1075-1083) the authors show the overlap in the mature retina between the MC specific Notch-reporter TP1:Venus and a GFAP transgenic line. Furthermore they use the GFAP-GFP line to verify the complete loss of MCs after DAPT treatment. In no instance in all of these papers is there any mention of MCs identified by other means, i.e. immunohistochemistry or other transgenic lines, where they are not labelled by the GFAP-GFP transgene. Hence, there can be no doubt that the GFAP-GFP transgenic line is a well-established line recognized for its ability to faithfully label MCs.*

*Secondly, even if there should be a problem with transplanted cells not expressing GFP in GFAP positive cells at the correct time-point then we would expect clones with no MCs or with more clones than predicted. Scenario one would be that the mature clone contains a MC that does not express GFP. This would mean that clones predicted to have one MC would have none, which would generate a significant amount of clones with 0 MC. As clearly showed in the data-set, this occurs extremely seldom. We would also see clones expected to produce two MCs that only contain one MC. This is also something we rarely see. In any case, as we also have the Ptf1a+ cells, this would worsen any correlation that we try to show rather than helping our argument. The second scenario is regarding the screening process; if the progenitors in the 24 hpf retina does not express GFP. This “unlabeled” donor cell would then be able to form a clone in conjunction to the clone of the screened cell resulting in more MCs and more Ptf1a+ cells than predicted by the screen. In the WT scenario we never came across a clone that contained more MCs than expected based on the screen. Also here, if one claim that our screen miss some of the transplanted cells then we would find a different correlation.*

*Thirdly, the Ptf1a positive cells are the ones used to find and define the clone. Here we show clearly that we can use the number of Ptf1a positive cells to verify that a clone has originated from one or two retinal progenitors. Hence, we have the initial screening, the number of Ptf1a positive cells, and the GFAP positive cells that all need to correlate in order to give us the results we present. Our thorough clonal analysis using H2B-GFP in Boije et al. 2015 show a large clonal dataset that is identical to the current regarding the distribution of Ptf1a. This shows that the screen for transplanted GFP positive cells in the GFAP-GFP transgenic line rendered the same results as the study where we screened for the ubiquitous neutral marker H2B-GFP.*

*Lastly, we used the Vsx2-GFP transgenic line to verify our findings. Here we get an identical correlation, which would suggests that there is no doubt of the results generated using the GFAP-GFP transgenic line.*

*To conclude, there is no evidence that the reporter is in any way flawed, even if it was it would prevent us from acquiring the correlation that we have, the comparison to our previous study verifies the screening method, and the use of a second transgenic marker verifies our results.*

● Another issue is that the authors estimate the clone size and the initial number of labeled RPCs in the unscreened clones based on the number of Ptf1a positive cells. This inference is crucial for all the downstream analysis in the WT and knockdown experiments. However, this reporter line only labels two cells types (amacrine and horizontal cells) that correspond to less than 30% of the total number of cells in the retina. Taking into consideration that the clones are highly variable in neuronal composition, it is not clear how the authors distinguish the technical variability (eg. number of cells labeled) from the intrinsic variability of the system to make such inferences in single clones. Moreover, it has been shown that the endpoints of the lineages are highly biased toward particular outcomes (Boije et al, 2015). Therefore, to test if the Müller glia fate assignment is in agreement with deterministic or stochastic models, the authors should have used a constitutively active reporter, as noted above, that labels the entire backbone of the lineage instead of a fraction of it that might be biased. Such a bias might explain the high prevalence of Muller glia in their analysis (148 out of 150 clones) when compared to previous lineage analysis performed (28 out of 396 clones) (He et al., 2012). 

*Response: We do not use the Ptf1a positive cells to estimate the clone size. We use Ptf1a-dsRed to identify the clone and to verify that we can directly correlate the number of start progenitors with the number of Ptf1a positive cells. We show that there is a clear bimodality in the distribution of Ptf1a cells originating from one or two transplanted cells. For the initial conclusions of the paper the Ptf1a positive cells are dispensable as the crucial correlation lies between the number of screened cells at 24 hpf and the number of MC at 72 hpf. The Ptf1a positive cells are then used to show this additional correlation allowing us to expand the data-set with additional clones that are not screened. Again, although the use of transgenic reporters and clonal variability might distort the data, this would only worsen the correlation and by no means provide us with the current result.*

*The variability in total clone size and the changes in morphant clones is thoroughly described in Boije et al. 2015 and is not relevant in this paper. The correlation is strictly between the origin progenitor and the MC, where Ptf1a is used in the morphants to infer the origin of the clone. If anything, we show here that the variability of neurons in WT clones is not affecting the MCs and that the correlation between the average clone size and the actual proportion of MCs found in the retina is close to identical.*

● The current manuscript is poorly written. For example, the authors did neither motivate nor conclude on the knockdown experiments. Further, considering that the results part is only three pages long, the discussion (and to some extend also introduction) seems extensive, mentioning many aspects that are not part of the current study. The same is true for Figure 4 which is only marginally related to the presented data.

*Response: We have tried to clarify why morphants were analyzed (in order to make the data-set comparable to our study regarding neural fate determination). As this is only a submitted as a short communication all the text has been minimized ahead of submission. However, the results are quite straight forwards and therefore easier to shorten while we wanted more space to introduce the question and conclude on the importance of the study. With this said, we have made an additional effort to shorten the mentioned paragraphs. The middle paragraph of the introduction has been moved and merged into the discussion. This shortens the manuscript and reduces repetition. Regarding figure 4; although one may claim that it is only “marginally related”, we believe that the summary of known neural lineages brings the current study into context for readers not familiar with some of the aspects.*
